# Age differences in neural distinctiveness during memory encoding, retrieval, and reinstatement

**DOI:** 10.1093/cercor/bhad219

**Published:** 2023-06-26

**Authors:** Claire Pauley, Malte Kobelt, Markus Werkle-Bergner, Myriam C Sander

**Affiliations:** Center for Lifespan Psychology, Max Planck Institute for Human Development, Lentzeallee 94, 14195 Berlin, Germany; Department of Psychology, Humboldt-Universität zu Berlin, Rudower Chaussee 18, 12489 Berlin, Germany; Department of Neuropsychology, Ruhr-Universität Bochum, Universitätsstraße 150, 44801 Bochum, Germany; Center for Lifespan Psychology, Max Planck Institute for Human Development, Lentzeallee 94, 14195 Berlin, Germany; Center for Lifespan Psychology, Max Planck Institute for Human Development, Lentzeallee 94, 14195 Berlin, Germany

**Keywords:** Aging, Episodic memory, fMRI, Neural dedifferentiation, Pattern similarity analyses

## Abstract

Robust evidence points to mnemonic deficits in older adults related to dedifferentiated, i.e. less distinct, neural responses during memory encoding. However, less is known about retrieval-related dedifferentiation and its role in age-related memory decline. In this study, younger and older adults were scanned both while incidentally learning face and house stimuli and while completing a surprise recognition memory test. Using pattern similarity searchlight analyses, we looked for indicators of neural dedifferentiation during encoding, retrieval, and encoding–retrieval reinstatement. Our findings revealed age-related reductions in neural distinctiveness during all memory phases in visual processing regions. Interindividual differences in retrieval- and reinstatement-related distinctiveness were strongly associated with distinctiveness during memory encoding. Both item- and category-level distinctiveness predicted trial-wise mnemonic outcomes. We further demonstrated that the degree of neural distinctiveness during encoding tracked interindividual variability in memory performance better than both retrieval- and reinstatement-related distinctiveness. All in all, we contribute to meager existing evidence for age-related neural dedifferentiation during memory retrieval. We show that neural distinctiveness during retrieval is likely tied to recapitulation of encoding-related perceptual and mnemonic processes.

## Introduction

Cognitive decline in neurologically healthy aging is frequently associated with a phenomenon called age-related neural dedifferentiation, the finding that neural distinctiveness is reduced in older adults compared with younger adults (for reviews, see Koen and Rugg 2019; Koen et al. 2020; Sommer and Sander 2022). In functional magnetic resonance imaging (fMRI) studies, neural distinctiveness is often operationalized by contrasting content-related neural activity between different visual categories (e.g. faces and houses) either using mean blood-oxygen-level-dependent (BOLD) signal (e.g. Park et al. 2004) or using multivoxel activity patterns (e.g. Hill et al. 2021). Recently, the impact of age-related neural dedifferentiation on episodic memory performance has received particular interest due to the significance of highly distinct neural representations for encoding and retrieving unique events. Several studies have established a relationship between low representational distinctiveness during *memory encoding* and poor memory performance in older adults (Zheng et al. 2018; Koen et al, 2019; Srokova et al. 2020), suggesting that the formation of well-defined, non-overlapping memory traces is important for memory performance.

Only a few studies have examined neural dedifferentiation during later stages of memory processing, with most of them assessing age differences in cortical reinstatement. The *cortical reinstatement hypothesis* suggests that cortical representations of information during memory encoding are recapitulated during memory retrieval as facilitated by the hippocampus (Norman and O’Reilly 2003; Johnson and Rugg 2007; for review, see Danker and Anderson 2010). Age-related declines in the precision of cortical reinstatement have been reported at both the item (St-Laurent et al. 2014; Bowman et al. 2019; Folville et al. 2020; Hill et al. 2021) and category (McDonough et al. 2014; Johnson et al. 2015; Abdulrahman et al. 2017; Bowman et al. 2019; Deng et al. 2021; Hill et al. 2021; Katsumi et al. 2021) representational levels (but, see Wang et al. 2016; Thakral et al. 2017; Thakral et al. 2019, for absent age effects, and Deng et al. 2021, for age-related hyperdifferentiation). A couple of studies have found that age differences in cortical reinstatement may be attributed to age differences in neural distinctiveness during encoding (Johnson et al. 2015; Hill et al. 2021). However, Trelle et al. (2020) demonstrated that reinstatement declined with age in a sample of older adults even after controlling for encoding-related distinctiveness. While there is substantial evidence suggesting that distinctive cortical reinstatement benefits memory performance (Ritchey et al. 2013; St-Laurent et al. 2014; Abdulrahman et al. 2017; Bowman et al. 2019; Hill et al. 2021), some studies have shown that cortical reinstatement can also occur even when individuals are unable to retrieve a particular memory (Thakral et al. 2017; Elward et al. 2021). Accordingly, cortical reinstatement alone may not be sufficient to facilitate successful memory retrieval, but functions in concert with other neural mechanisms, including retrieval-related hippocampal activity. Although early neurobiological models posit that, during retrieval, the hippocampus facilitates cortical reinstatement via pattern completion (Marr 1971; McClelland et al. 1995), implying that hippocampal activity and cortical reinstatement might explain similar sources of variance in memory outcomes, recent evidence has suggested that these 2 mechanisms predict memory performance (at least partially) independently from one another (Ritchey et al. 2013; Trelle et al. 2020; Hill et al. 2021).

Beyond reinstatement, which, per definition, is a process closely tied to encoding, recent evidence has suggested that neural representations supporting memory retrieval can also differ from those initially formed during memory encoding due to spatial transformations (for review, see Favila et al. 2020). Several studies have shown that neural patterns of retrieved information were spatially distinct from the neural patterns representing the same information during encoding (Xiao et al. 2017; Favila et al. 2018; Srokova et al. 2022). These findings support the long-standing idea that memory retrieval is not simply a reproductive process, but rather more of a constructive process (Schacter et al. 1998) that draws on cognitive processes (and neural correlates) unique to retrieval, such as retrieval mode, effort, and orientation (see Rugg and Wilding 2000). So far, the literature on neural dedifferentiation has largely ignored the possibility that neurally distinctive patterns support memory retrieval, may be susceptible to age-related decline, and may not only reflect reinstated encoding patterns. First evidence comes from a study by Srokova et al. (2022), who demonstrated that both younger and older adults exhibited a systematic shift in the locus of neural distinctiveness, in which the peak of neural distinctiveness was observed more anteriorly during memory retrieval as compared with memory encoding. Importantly, the magnitude of this so-called anterior shift was greater in older adults, suggesting that age may amplify spatial transformations between encoding and retrieval.

Thus, investigations of age-related neural dedifferentiation during memory retrieval may be more comprehensive when considering memory retrieval also independently of reinstatement effects. To date, few studies have examined this (Dulas and Duarte 2012; St-Laurent et al. 2014; Johnson et al. 2015; St-Laurent and Buchsbaum 2019; Hill et al. 2021). All studies found evidence for an age-related decline in neural distinctiveness during retrieval. However, results were mixed with regard to the role of age differences during encoding for the observed effects. Whereas St-Laurent et al. (2014) reported that age differences in neural distinctiveness during retrieval could not be attributed to age differences during encoding, Johnson et al. (2015) showed that age differences in neural distinctiveness during retrieval were eliminated when controlling for age differences during encoding. In a follow-up analysis, St-Laurent and Buchsbaum (2019) further found that retrieval-related distinctiveness likely reflected precise reinstatement in younger adults, but not in older adults, suggesting that older adults’ retrieval-based representations are less tied to their corresponding encoding representations.

Altogether, a comprehensive assessment of age differences in neural distinctiveness across different stages of memory processing, with a focus on the relation between neural distinctiveness and performance, is currently missing. Here, we collected fMRI data while a group of younger and older adults learned images of faces and houses and subsequently performed an old/new recognition memory test. Using exploratory pattern similarity searchlight analyses, we looked for regions expressing high neural distinctiveness (operationally termed *specificity* throughout the Methods and Results) during memory encoding and retrieval as well as in encoding–retrieval reinstatement (both at the category and individual item levels). We were particularly interested in whether we would find evidence of age-related neural dedifferentiation across different memory phases and whether neural distinctiveness would be associated with memory performance. We expected older adults to demonstrate reduced distinctiveness during all memory phases, particularly in visual processing regions. We additionally examined whether age differences in retrieval- and reinstatement-related distinctiveness could be explained by age differences in encoding-related distinctiveness. We looked at whether neural distinctiveness was associated with memory performance both across individuals and within persons, expecting to find significant relationships at both levels of analysis. Finally, we examined how item-level reinstatement and hippocampal activity related to memory outcomes (hit or miss) at the individual trial level.

## Materials and methods

Encoding and retrieval data from this project were previously reported in 2 papers (Kobelt et al. 2021; Pauley et al. 2022) that were later retracted by the authors due to a preprocessing error. For the retracted manuscripts as well as comparison reports with the corrected findings, please see https://osf.io/t8dpv/ and https://osf.io/7n3mz/.

### Participants

Data were collected from a total of 76 healthy adults. Participants were recruited within 2 age groups: younger adults (18–27 years, N = 39) and older adults (64–76 years, N = 37). Two participants were excluded due to too much motion in the scanner (1 younger adult and 1 older adult), 3 were excluded due to memory performance below chance level (2 younger adults and 1 older adult), and 1 younger adult was excluded due to poor MRI data quality. The final sample consisted of 35 younger adults (*M* (*SD*) age = 22.3 (2.7) years, 16 females, 19 males) and 35 older adults (*M* (*SD*) age = 70.6 (2.4) years, 19 females, 16 males). Participants were screened via telephone for mental and physical illness, metal implants, and current medications. Additionally, all older adults were screened using the Mini-Mental State Examination (Folstein et al. 1975) and all exceeded the threshold of 26 points. The study was approved by the ethics committee of the German Society for Psychological Research (DGPs) and written informed consent was obtained from each participant at the time of the study.

### Stimuli

Stimuli were comprised of 300 grayscale images belonging to 3 different categories: 120 neutral faces (adapted from the FACES database; Ebner et al. 2010), 120 houses (some adapted from DC Park et al. 2004, and some obtained online), and 60 phase-scrambled images (30 faces and 30 houses, constructed from randomly selected face and house images) serving as control stimuli. An additional image from each category was selected to serve as target stimuli for the encoding target-detection task. All nontarget face and house images were randomly divided into 2 sets of 120 images (60 faces and 60 houses). One stimulus set was presented during both encoding and recognition (old images) and the other set was presented only during recognition (new images). The same stimulus sets were used for all participants.

### Paradigm

**Fig. 1:**
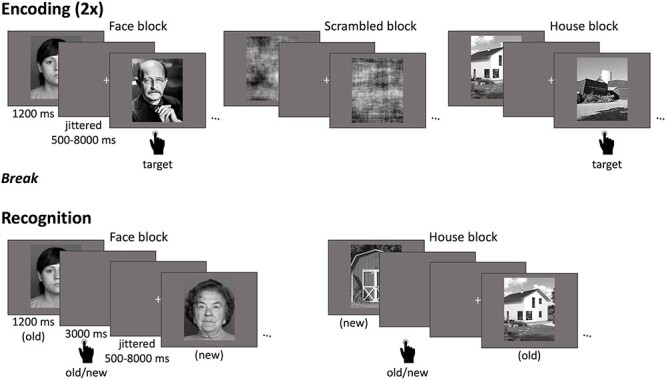
Face-house task design. This fMRI paradigm comprised an incidental encoding phase (top) and a surprise recognition test (bottom). During encoding, 2 identical runs of face, house, and phase-scrambled images (not assessed here) were presented in a block design with 9 stimulus blocks each (3 alternating blocks from each stimulus category). Each block had 21 trials (20 exemplars of the respective category and 1 pre-learned target stimulus). Participants were instructed to press a button when a target stimulus appeared. During the recognition test, 6 alternating face and house blocks were presented with 40 trials each (20 old trials from encoding and 20 new trials). Participants indicated via button press whether each image was old or new.

The following paradigm was part of a larger study spanning 2 days of data collection. This study focuses only on the face-house task, which comprised an incidental encoding phase and a surprise recognition test (in line with many prior aging studies; see Fraundorf et al. 2019, for a meta-analysis), both conducted inside the fMRI scanner on the same day with a delay of approximately 30 min. (see Fig. 1). The encoding phase consisted of 2 identical runs each with 9 stimulus blocks. In order to ensure the participants were paying attention to the stimuli, they were asked to perform a target-detection task in which they pressed a button when 1 of 3 pre-learned target images was presented. Stimuli were randomly distributed into the blocks such that each block contained 20 images of a single category (faces, houses, or phase-scrambled) as well as a category-matched target image. The block order was alternating and counterbalanced across participants, always starting with either a face or house block. The stimulus order within each block was pseudo-randomized with the condition that the target image was not presented in either the first 4 or last 4 trials of a block. Due to a technical problem, the same stimulus order was used for all participants who started with a face block and for 36 of the participants starting with a house block. Prior to the encoding phase, participants completed 5 practice trials of each stimulus category, including each of the target stimuli, to verify that they understood the target-detection task. The nontarget training stimuli were excluded from the main experiment. Since the 2 encoding runs were identical, participants were exposed to each stimulus twice during the encoding phase. Phase-scrambled images were not used in any subsequent analyses in this project. Stimuli were presented for 1200 ms and separated by a fixation cross with a jittered duration between 500 and 8000 ms. In total, the encoding phase lasted approximately 22 min.

Following encoding, participants remained in the scanner briefly while structural scans were collected (see below). Then, they had a short break outside the scanner while they received instructions for the recognition test. They then returned to the scanner to complete the recognition test. The recognition test consisted of 6 blocks in total, alternating between 3 face and 3 house blocks and was divided into 2 runs of 3 blocks each. Each block contained 20 old images (seen during encoding) and 20 new images of the same stimulus category. For each trial, participants were asked whether the image was old or new, which they indicated via button press. The stimulus order was pseudo-randomized such that no more than 3 old or new images were presented consecutively. Due to a technical problem, the same stimulus order was used for 13 participants who started with a face block and 14 participants who started with a house block. Stimuli were presented for 1200 ms and followed by a gray screen for 3000 ms in which participants could give their response. Fixation crosses separated the trials with jittered durations between 500 and 8000 ms. In total, the recognition task lasted approximately 26 min.

### fMRI data acquisition and preprocessing

Brain imaging was acquired with a Siemens Magnetom TrioTim 3T MRI scanner with a 32-channel head-coil. Functional images were collected using an echo planar imaging sequence during both the encoding and recognition phases in 2 runs each. Each encoding run consisted of 270 volumes and each recognition run consisted of 372 volumes (voxel size = 3 × 3 × 3 mm^3^; slice gap = 0.3 mm; TR = 2 s; TE = 30 ms). The first 3 volumes of each run were dummy volumes and were excluded prior to preprocessing. Following the encoding phase, a T1-weighted (T1w) magnetization prepared rapid acquisition gradient echo (MPRAGE) pulse sequence image was acquired (voxel size = 1 × 1 × 1 mm^3^; TR = 2.5 ms; TE = 4.77 ms; flip angle = 7°; TI = 1.1 ms). Additionally, turbo spin-echo proton density images, diffusion tensor images, and fluid attenuation inversion recovery images were collected, but not included in the following analyses. Experimental stimuli, which participants viewed via a mirror mounted on the head-coil, were projected using the Psychtoolbox (Psychophysics Toolbox) for MATLAB (Mathworks Inc., Natick, MA).

MRI data were organized according to the Brain Imaging Data Structure (BIDS) specification (Gorgolewski et al. 2016) and preprocessed using *fMRIPrep* (version 1.4.0; Esteban et al. 2019) with the default settings. The T1w image was corrected for intensity nonuniformity, skull-stripped, and spatially normalized to the *ICBM 152 Nonlinear Asymmetrical template version 2009c* through nonlinear registration. Functional images were motion-corrected, slice-time corrected, and co-registered to the normalized T1w reference image. As mentioned in Section 2.1, 2 participants were excluded due to excessive motion in the scanner. Excessive motion was defined as having multiple motion artifacts within a given scanner run resulting in framewise displacements greater than the size of the voxel (3 mm; see Power et al. 2012). Finally, functional images were resampled to 2 mm isotropic voxels in order to enhance the signal-to-noise ratio (Dimsdale-Zucker and Ranganath 2018).

### Behavioral data analyses

Behavioral analyses were performed using custom MATLAB scripts. Recognition memory performance (*Pr*) was measured as the difference between the hit rate (proportion of correctly identified old stimuli) and the false alarm rate (proportion of new stimuli incorrectly identified as old stimuli; Snodgrass and Corwin 1988). Participants with *Pr* less than zero, indicating a higher probability of responding “old” to new stimuli as opposed to old stimuli, were excluded from further analyses (see also Section 2.1). Dependent-samples *t*-tests were used to determine whether memory performance exceeded chance level. A 2-way mixed factorial analysis of variance (ANOVA) was used to assess recognition memory performance for age differences and differences related to stimulus type (face vs. house). Age differences in response bias were assessed with independent-samples *t*-tests comparing the hit rates and false alarm rates across age groups.

### Pattern similarity searchlight analyses

In order to perform pattern similarity analyses, a general linear model was performed for each trial in both encoding and recognition, including 1 trial-specific regressor, 1 regressor for all other trials within the same run, and 6 motion regressors (Mumford et al. 2012). Trial regressors were modeled as 1.2 s duration boxcar functions convolved with a canonical hemodynamic response function. Pattern similarity analyses were based on the resulting β weights for each trial. Pattern similarity was only assessed between trials from different runs to control for time-dependent correlations in the hemodynamic responses (Dimsdale-Zucker and Ranganath 2018) and was measured as Fisher *z*-transformed Pearson correlations. Searchlight similarity analyses were conducted using modified scripts from the MATLAB toolbox for representational similarity analysis (Nili et al. 2014) and with 8-mm-radius spherical searchlights (analyses were replicated using 4-mm- and 12-mm-radius searchlights).

**Fig. 2:**
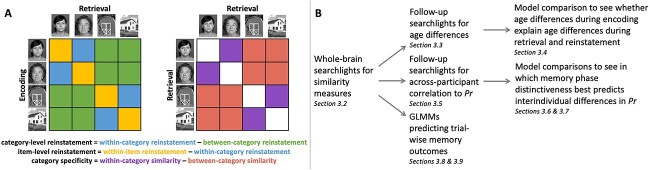
Illustration of searchlight similarity measures calculated during encoding–retrieval reinstatement (A—left) and during recognition (A—right). Note that the measure of category specificity during encoding mirrors the measure during recognition. Flow-diagram demonstrating the analysis pipeline and corresponding results sections (B).

Several searchlight similarity measures were computed (see Fig. 2). For all measures, we first ran searchlights looking for the measures of interest across all participants, followed by post-hoc searchlights for age differences and correlation to memory performance within the regions in which we identified a main effect. First searching for our measures of interest across all participants was necessary in order to establish neural regions demonstrating the baseline effects of interest. We ran this initial analysis on the whole sample as opposed to only within younger adults in order to have an age-fair comparison and avoid introducing bias into our subsequent search for age differences (see Supplements showing comparable results when performing the main analyses within each age group independently).

First, in order to identify brain regions demonstrating high category specificity across all participants during *memory encoding* and *memory recognition*, we compared within-category similarity to between-category similarity for both faces and houses separately. For each stimulus, within-category similarity was calculated as the averaged across-voxel correlation of the activity pattern in response to the stimulus to the activity patterns in response to all other stimuli from the same category (e.g. mean similarity of the activity in response to a face stimulus to that of all non-identical face stimuli). For each participant, within-category similarity was then averaged across all stimuli within each category. Between-category similarity was calculated as the averaged across-voxel correlation of the stimulus’ activity pattern to the activity patterns of all stimuli from the other category (e.g. mean similarity of the activity in response to a face stimulus to that of all house stimuli). Between-category similarity was then averaged across all stimuli for each participant. Within- and between-category similarity were assessed in a searchlight centered on each voxel in the brain and the difference was calculated, resulting in a whole-brain map of category specificity for faces and a whole-brain map of category specificity for houses in each participant and for each memory phase.

Next, we were interested in searching for brain regions exhibiting *encoding–retrieval reinstatement* both at the category level and at the individual stimulus level. In order to assess category-level reinstatement, we compared within-category reinstatement to between-category reinstatement. Within-category reinstatement was calculated as the mean pattern similarity of all stimuli from a given category during encoding to all stimuli from the same category during recognition. Within-item reinstatement correlations (i.e. the similarity of a given stimulus’ activity pattern during encoding to the activity pattern of the same stimulus during recognition) were excluded from the measure of within-category reinstatement. Between-category reinstatement was similarly calculated as the mean pattern similarity of all stimuli from a given category during encoding to all stimuli from the other category during recognition. Within- and between-category reinstatement were then averaged across all stimuli in each searchlight and the difference in each voxel was computed, resulting in a whole-brain map of category-level reinstatement for each participant. A whole-brain map of item-level reinstatement was created for each participant by calculating the voxel-wise difference between within-item reinstatement and within-category reinstatement. For both category- and item-level reinstatement, similarity values were calculated between recognition and each encoding run individually, then the reinstatement similarity values were averaged across encoding run.

For each searchlight similarity measure, nonparametric, cluster-based, random permutation analyses adapted from the FieldTrip toolbox (Oostenveld et al. 2011) were used to identify brain regions demonstrating significant effects across all participants (e.g. for the high category specificity measures, regions demonstrating greater within-category similarity than between-category similarity). First, dependent-samples *t*-tests were conducted within each voxel. Adjacent voxels with significance values lower than a threshold of *p* < 0.005 were grouped into clusters. The sum of all *t* statistics of the voxels included in each cluster was defined as the cluster test statistic. The Monte Carlo method was used to determine whether a cluster was significant by comparing the cluster test statistic to a reference distribution of *t* statistics across 1000 permutations. Each *t* statistic in the reference distribution was created by randomly reallocating the 2 conditions and calculating the cluster test statistic based on this random reallocation. Clusters were considered significant under a threshold of *p* < 0.05 and if they contained at least 10 voxels.

### Assessing age differences in searchlight similarity analyses

We were also interested in searching for brain regions demonstrating age differences in each of our searchlight similarity measures. For our age comparison analyses, we limited the search space to the regions identified during the whole-group analyses (i.e. age differences were only assessed in regions demonstrating an effect across all participants). Since some of the clusters identified during the whole-group analyses were fairly large (>8000 voxels), we again used nonparametric, cluster-based, random permutation analyses to search these clusters for regions exhibiting age differences. First, independent-samples *t* tests were conducted within each voxel comparing younger and older adults on each searchlight similarity measure described previously. Adjacent voxels with significance values lower than a threshold of *p* < 0.005 were grouped into clusters and the sum of all *t* statistics of the voxels included in each cluster was defined as the cluster test statistic. The Monte Carlo method was again used to determine whether a cluster was significant. In this case, the reference distribution was created by removing the younger and older adult labels and randomly assigning participants to each age group across 1000 permutations.

In order to determine whether age differences in recognition category specificity or category-level reinstatement could be attributed to age-related variability during initial encoding, we additionally ran 2 model comparisons, one predicting recognition category specificity and one predicting category-level reinstatement. For each model comparison, one variable block was computed using age group as a single predictor and the other block used both age group and encoding category specificity as predictors. The models were compared using the *anova()* function in *R*.

### Relating searchlight similarity measures to memory performance

We further assessed the relationship between our searchlight similarity measures and interindividual differences in memory performance. Therefore, permutation testing was performed again for each cluster identified during the whole-group analyses. For this, regressions were conducted predicting memory performance from the searchlight similarity measure in each voxel (i.e. recognition category specificity, category-level reinstatement, item-level reinstatement). As described previously, adjacent voxels below the threshold of *p* < 0.005 were grouped into clusters, the cluster test statistic was calculated, and the Monte Carlo method determined the significance of each cluster across 1000 permutations. In order to derive the correlation coefficient for significant clusters to better understand the relationships, we averaged the respective searchlight similarity measure across all voxels within the cluster for each participant and correlated this with *Pr* across participants using Pearson correlations across all participants, within younger adults, and within older adults (these can be found in Table 3). Clusters identified during face analyses were correlated with *Pr* for faces and clusters identified during house analyses were correlated with *Pr* for houses. Age differences in correlation coefficients between younger and older adults were assessed using *z* tests.

Additionally, we were interested in whether recognition category specificity or category-level reinstatement was better at tracking interindividual variability in memory performance. To this end, we computed 2 linear model comparisons predicting *Pr*. For the first model comparison, one variable block was computed using age and recognition category specificity (i.e. Pr ~ Age^*^RecSpec) and the other block added the interaction between age and category-level reinstatement (i.e. Pr ~ Age^*^RecSpec + Age^*^ReinSpec). The second model comparison included age and category-level reinstatement as predictors in one block and additionally the interaction between age and recognition category specificity in the other block. In this way, we were able to determine whether recognition category specificity or category-level reinstatement better explained memory-related variance.

In a final step, we wanted to know whether encoding category specificity explained any additional memory-related variance on top of both recognition category specificity and category-level reinstatement. Therefore, we computed a linear model comparison in which the first block predicted memory performance from recognition category specificity and category-level reinstatement (i.e. Pr ~ Age^*^RecSpec + Age^*^ReinSpec) and the second block added encoding category specificity to the model (i.e. Pr ~ Age^*^RecSpec + Age^*^ReinSpec + Age^*^EncSpec). We further asked whether recognition category specificity or category-level reinstatement explained any variance in memory performance not already accounted for by encoding category specificity. Thus, we computed 2 additional model comparisons in the same manner as described in the previous paragraph, in which the first block included only encoding category specificity and the second block added either recognition category specificity or category-level reinstatement.

### Trial-wise mixed effects analyses

In order to relate our findings of category specificity to memory performance at the within-person level, we additionally performed a series of generalized linear mixed-effects models predicting memory outcomes from trial-wise category specificity within each of the clusters identified by the searchlight similarity analyses (as defined in Section 2.6). For each model, binary recognition memory outcomes (hit or miss) were used as the dependent variable and age, response bias, and category specificity (operationalized as the difference between the similarity of the given item to all other items of the same stimulus category and the similarity between that item and all items of the other stimulus category) were used as independent variables. The interaction between age and category specificity was also included in the models. The models were analyzed using the *R* function *glmer* from the lme4 package with the following formula: Memory ~ Age^*^CatSpec + RespBias + (1 + CatSpec | Subject).

Recent work has pointed to the significance of both reinstatement and hippocampal activity in predicting within-person variability in memory performance in both younger and older adults (Trelle et al. 2020; Hill et al. 2021). In order to investigate this further, we performed generalized linear mixed-effects models to determine whether item- or category-level reinstatement specificity or trial-wise hippocampal activity were successful predictors of *intra*individual differences in memory performance. To measure trial-wise hippocampal activity, we used a bilateral hippocampal mask defined by the automatic anatomic labeling atlas. For each participant, we averaged across all beta weights within the hippocampal mask for every trial and z-transformed across trials within each participant. We performed a separate model for each cluster resulting from our item-level reinstatement searchlight similarity analyses. For each model, binary recognition memory outcomes (hit or miss) were used as the dependent variable and age, response bias, hippocampal activity, item-level reinstatement (operationalized as the difference between the similarity of an item to itself across encoding and recognition and the mean similarity of the same item to all other items within the same stimulus category), and category-level reinstatement were used as independent variables. The interactions between age and hippocampal activity as well as age and item- and category-level reinstatement were included in the models. The following formula was used for each model: Memory ~ Age^*^Hipp + Age^*^ItemRein + Age^*^CatRein + RespBias + (1 + Hipp + ItemRein + CatRein | Subject).

## Results

### Behavioral results

We first checked for age differences in memory performance (i.e. *Pr* = hit rate—false alarm rate). Memory performance exceeded chance level in both younger (*t*(34) = 11.88, *p* < 0.001) and older adults (*t*(34) = 9.25, *p* < 0.001). A mixed factorial ANOVA revealed no interaction between age group and stimulus type on memory performance (*F*(1,68) = 0.09, *p* = 0.77). Additionally, memory performance in terms of *Pr* did not differ between age groups (*M*_younger_ = 0.24, *SD*_younger_ = 0.12, *M*_older_ = 0.19, *SD*_older_ = 0.12, *F*(1,68) = 3.20, *p* = 0.08) or between face and house stimuli (*F*(1,68) = 2.83, *p* = 0.10). However, older adults responded “old” more often than younger adults to both old stimuli (i.e. hits; *M*_younger_ = 0.50, *SD*_younger_ = 0.14, *M*_older_ = 0.60, *SD*_older_ = 0.13, *t*(68) = −3.27, *p* = 0.002) and new stimuli (i.e. false alarms; *M*_younger_ = 0.26, *SD*_younger_ = 0.11, *M*_older_ = 0.42, *SD*_older_ = 0.13, *t*(68)  = −5.42, *p* < 0.001).

### Category specificity during encoding, retrieval and reinstatement in occipital and ventral visual regions

**Fig. 3:**
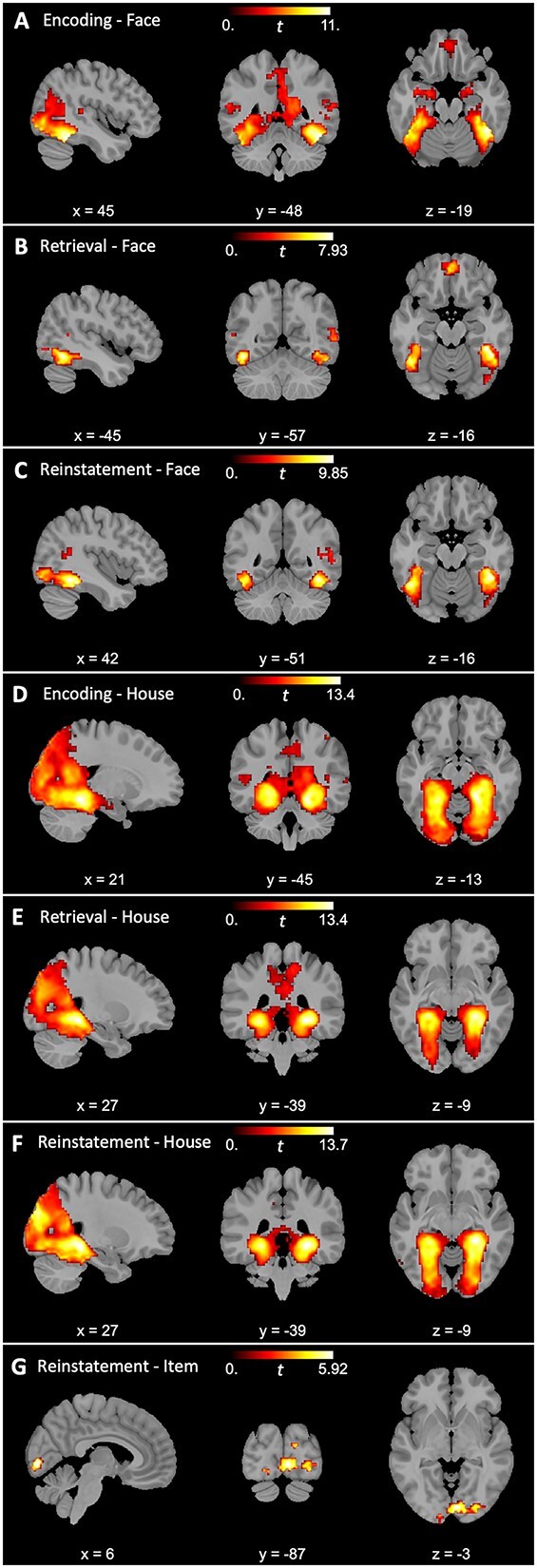
Regions demonstrating category specificity during encoding, retrieval, and reinstatement for both faces (A, B, C) and houses (D, E, F). Regions demonstrating item-level reinstatement specificity for houses (G).

We first searched for regions exhibiting greater within-category similarity than between-category similarity in the whole sample of participants for both faces and houses during encoding. We identified 4 clusters demonstrating category specificity for faces in ventral visual, frontal, and medial temporal regions (*p*s < 0.01; see Table 1 and Fig. 3) and 2 clusters demonstrating category specificity for houses in ventral visual and occipital regions (*p*s < 0.05).

**Table 1:** Clusters identified by searchlight similarity analyses revealing high category specificity during encoding, retrieval, and reinstatement.

			Peak MNI				
Searchlight	Regions	H	X	Y	Z	Peak *t*	*k*
**Face** **Encoding**	Middle temporal gyrus, fusiform gyrus, precuneus	B	45	−49	−19	11.04	5003
***(Category)***	Medial orbitofrontal gyrus, rectus	B	6	57	−13	5.05	255
	Hippocampus, putamen, amygdala, pallidum, fusiform gyrus	L	−22	−10	−6	5.20	247
	Amygdala, hippocampus, putamen, pallidum	R	18	−7	−9	5.29	166
**Face** **Retrieval** ***(Category)***	Middle temporal gyrus, superior temporal gyrus, fusiform gyrus, inferior occipital gyrus, inferior temporal gyrus	R	42	−43	−19	7.85	566
	Fusiform gyrus, inferior temporal gyrus, inferior occipital gyrus	L	−46	−58	−16	7.93	233
	Rectus, medial orbitofrontal gyrus	B	−1	54	−16	5.84	76
	Middle temporal gyrus, superior temporal gyrus	L	−55	−52	14	4.85	64
**Face Reinstatement** ***(Category)***	Middle temporal gyrus, fusiform gyrus, inferior temporal gyrus, inferior occipital gyrus, superior temporal gyrus	R	42	−52	−16	9.85	659
	Fusiform gyrus, inferior occipital gyrus, inferior temporal gyrus	L	−46	−58	−16	9.39	388
**House** **Encoding**	Middle occipital gyrus, precuneus	B	21	−46	−13	13.40	11,148
***(Category)***	Caudate	R	3	−1	7	4.41	75
**House** **Retrieval** ***(Category)***	Middle occipital gyrus, precuneus, parahippocampal gyrus	B	27	−40	−9	13.37	8018
**House Reinstatement** ***(Category)***	Middle occipital gyrus, lingual gyrus, precuneus, parahippocampal gyrus	B	27	−40	−9	13.68	8630
	Middle cingulate cortex	B	−1	−19	27	4.51	81
**House Reinstatement**	Calcarine cortex, cuneus, lingual gyrus, inferior occipital cortex	B	6	−88	−3	5.92	196
***(Item)***	Fusiform gyrus, inferior occipital cortex, lingual gyrus, middle occipital gyrus, calcarine cortex, superior occipital gyrus	L	−31	−76	−9	5.00	83

H = hemisphere; *k* = cluster size in voxels.

During recognition, our searchlight similarity analysis revealed 4 clusters demonstrating category specificity for faces in ventral visual, temporal, and frontal regions (*p*s < 0.05). We additionally identified one large cluster demonstrating category specificity for houses in ventral visual and occipital regions (*p* < 0.001).

Next, we searched for regions demonstrating encoding–retrieval reinstatement at the category level for faces and houses. We found 2 clusters demonstrating category-level reinstatement for faces in temporal and occipital regions (*p*s < 0.001) and 2 clusters demonstrating category-level reinstatement for houses in occipital regions (*p* < 0.04).

We also searched for regions demonstrating encoding–retrieval similarity at the item level for both faces and houses. These item-level reinstatement searchlight similarity analyses yielded 2 clusters in occipital regions for houses (*p*s < 0.02), but no significant clusters for faces (*p*s > 0.08).

### Age differences in category specificity

**Fig. 4:**
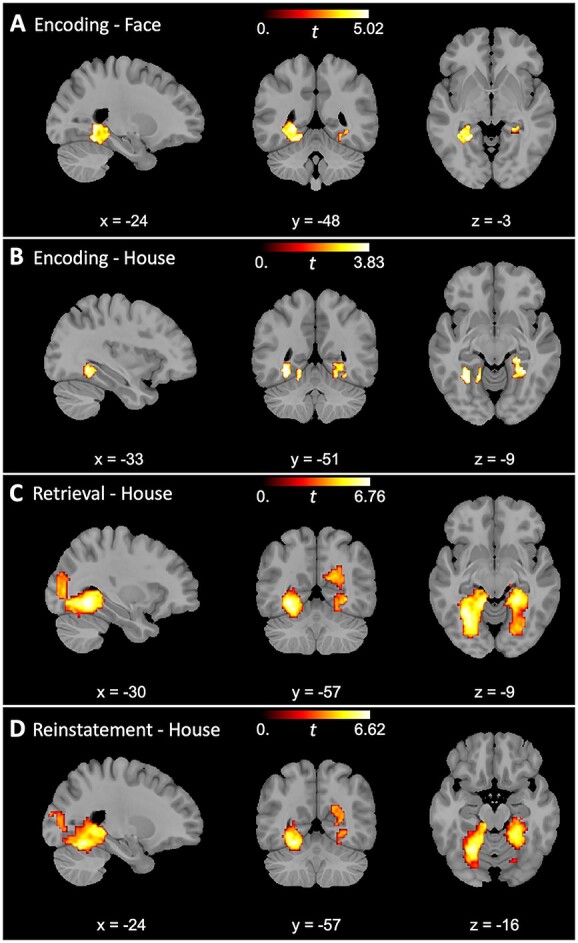
Age differences in category specificity. Younger adults demonstrated greater category specificity (within-category similarity > between-category similarity) than older adults during encoding for both faces (A) and houses (B) as well as during recognition (C) and reinstatement (D) for house stimuli.

Previous findings reveal clear age deficits in encoding-related specificity (Zheng et al. 2018; Koen et al, 2019; Srokova et al. 2020), and reinstatement specificity (St-Laurent et al. 2014; Abdulrahman et al. 2017; Bowman et al. 2019; Trelle et al. 2020; Hill et al. 2021), particularly in occipital and temporal regions, but age deficits in retrieval-related specificity are relatively less documented (St-Laurent et al. 2014; Johnson et al. 2015). Accordingly, we used cluster permutation analyses to test for age differences in category specificity, limiting the search space to regions identified by the whole-group analyses. During encoding of faces, younger adults exhibited greater category specificity than older adults in 3 clusters within bilateral ventral visual cortices (*p*s < 0.01; see Table 2 and Fig. 4). No age differences were identified in category specificity for faces during either recognition or reinstatement (all clusters were smaller than 10 voxels). In the analyses for age differences in category specificity for houses, 2 clusters were identified during encoding (*p*s < 0.01), 2 clusters during recognition (*p*s < 0.001), and 3 clusters in category-level reinstatement for houses (*p*s < 0.05) in bilateral ventral visual cortices. No age differences were identified in item-level house reinstatement.

**Table 2:** Clusters revealing greater category specificity in younger adults than in older adults.

			Peak MNI				
**Searchlight**	**Regions**	**H**	**X**	**Y**	**Z**	**Peak *t***	***k***
**Face** **Encoding**	Fusiform gyrus, lingual gyrus, parahippocampal gyrus	L	−25	−49	−3	5.02	174
***(Category)***	Fusiform gyrus, parahippocampal gyrus, hippocampus	R	33	−40	−9	4.28	122
	Superior occipital gyrus, precuneus, cuneus	R	27	−67	24	4.15	52
**House Encoding**	Fusiform gyrus, lingual gyrus, parahippocampal gyrus, cerebellum	L	−34	−52	−9	3.83	131
***(Category)***	Fusiform gyrus, lingual gyrus, parahippocampal gyrus, cerebellum	R	27	−37	−16	3.78	109
**House Retrieval**	Middle occipital gyrus, fusiform gyrus, lingual gyrus, cerebellum	L	−31	−58	−9	6.76	1034
***(Category)***	Fusiform gyrus, lingual gyrus, middle occipital gyrus, cerebellum, superior occipital gyrus, parahippocampal gyrus, precuneus	R	21	−43	−13	6.75	1029
**House Reinstatement**	Middle occipital gyrus, fusiform gyrus, lingual gyrus, cerebellum	L	−24	−58	−16	6.62	867
***(Category)***	Fusiform gyrus, cerebellum, lingual gyrus, parahippocampal gyrus, calcarine cortex, superior occipital gyrus, hippocampus	R	24	−34	−6	6.50	708
	Fusiform gyrus, lingual gyrus, cerebellum	R	24	−73	−9	3.88	39

H = hemisphere; *k* = cluster size in voxels.

### Age-related variance in recognition category specificity and category-level reinstatement attributed to encoding-related specificity

We additionally investigated whether encoding category specificity predicted interindividual differences in either recognition category specificity or category-level reinstatement using 2 linear model comparisons. The first model comparison revealed that adding encoding category specificity as a predictor improved the model fit on recognition-related specificity (*R^2^* = 0.43) as compared with age group alone (*R^2^* = 0.17; *F*(66) = 16.07, *p* < 0.001). The second model comparison also revealed that adding encoding-related specificity significantly improved the model fit on category-level reinstatement (*R^2^* = 0.77) as compared with age group alone (*R^2^* = 0.19; *F*(66) = 84.53, *p* < 0.001). After adding encoding category specificity, age group was no longer a significant predictor in either model (*p*s > 0.56). These findings suggest that age differences in both recognition category specificity and category-level reinstatement are largely attributable to age differences in encoding category specificity.

### Category specificity predicts interindividual differences in memory performance

**Fig. 5:**
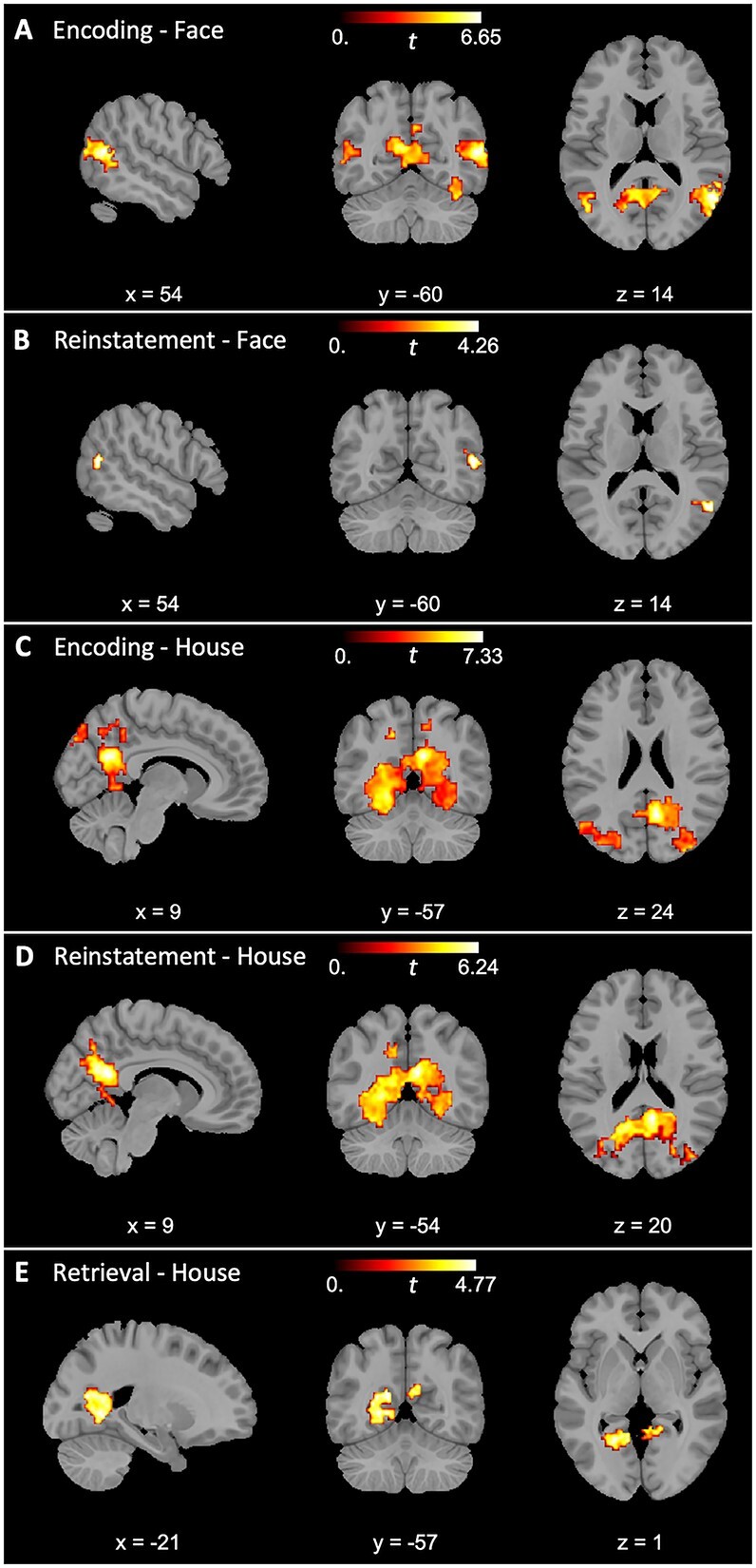
Regions indicating a relationship between category-level specificity and memory performance for faces during encoding (A), reinstated faces (B), houses during encoding (C), reinstated houses (D), and houses during recognition (E).

Here, we asked whether category specificity in the regions identified by the whole-group analyses was linked to memory performance. In temporal cortices, we identified 7 clusters demonstrating a positive relationship between memory performance for faces during encoding (*p*s < 0.05; see Table 3 and Fig. 5) and an additional 2 clusters in category-level reinstatement (*p*s < 0.001). However, we did not identify any regions demonstrating a relationship between memory performance and face specificity during recognition (all clusters had fewer than 10 voxels). In occipital and ventral visual regions, we also found a cluster revealing a positive relationship between memory performance and category specificity during encoding for houses (*p* < 0.001), one cluster in category-level reinstatement (*p* < 0.001), and 2 clusters during recognition (*p*s < 0.001). We did not identify any clusters revealing a relationship between memory performance and item-level reinstatement specificity for houses.

**Table 3:** Clusters in which category specificity correlates with memory performance.

			Peak MNI							
Searchlight	Regions	H	X	Y	Z	*k*	*r* (All)	*r* (YA)	*r* (OA)	*z*
**Face** **Encoding**	Calcarine cortex, lingual gyrus, precuneus, cuneus	B	21	−31	−13	470	0.63^*^	0.69^*^	0.56^*^	0.85
***(Category)***	Middle temporal gyrus, inferior occipital gyrus, middle occipital gyrus, fusiform gyrus, cerebellum	L	−49	−67	11	265	0.58^*^	0.66^*^	0.46^*^	1.18
	Middle temporal gyrus	R	54	−61	14	261	0.61^*^	0.68^*^	0.52^*^	0.99
	Fusiform gyrus, inferior occipital gyrus, cerebellum	R	39	−49	−29	198	0.56^*^	0.59^*^	0.55^*^	0.23
	Hippocampus, amygdala, putamen, pallidum	L	−22	−7	−22	125	0.56^*^	0.75^*^	0.48^*^	1.76
	Amygdala, hippocampus, pallidum	R	24	−7	−13	90	0.51^*^	0.48^*^	0.55^*^	−0.37
	Fusiform gyrus, inferior temporal gyrus	L	−40	−40	−16	57	0.47^*^	0.53^*^	0.39^*^	0.72
**Face**	Middle temporal gyrus	R	54	−61	14	34	0.44^*^	0.59^*^	0.23	1.77
**Reinstatement *(Category)***	Inferior temporal gyrus	L	−46	−40	−16	14	0.44^*^	0.52^*^	0.33^*^	0.94
**House** **Encoding *(Category)***	Middle occipital gyrus, precuneus, lingual gyrus, superior parietal gyrus	B	9	−58	24	3480	0.66^*^	0.83^*^	0.42^*^	3.02^*^
**House Retrieval**	Calcarine cortex, lingual gyrus, precuneus	L	−22	−58	1	228	0.47^*^	0.54^*^	0.44^*^	0.56
***(Category)***	Precuneus, lingual gyrus, calcarine cortex	R	9	−58	17	126	0.42^*^	0.57^*^	0.13	2.09^*^
**House Reinstatement *(Category)***	Precuneus, lingual gyrus, calcarine cortex, fusiform gyrus	B	9	−55	21	2424	0.61^*^	0.82^*^	0.24	3.65^*^

H = hemisphere; *k* = cluster size in voxels; *r* = Pearson correlation coefficient across all participants (All), younger adults only (YA), and older adults only (OA); ^*^p < 0.05; *z* = *z* value difference between *r* for YA and OA.

### Category-level reinstatement tracks interindividual variability in memory performance better than recognition category specificity

We additionally investigated whether either recognition category specificity or category-level reinstatement was better at explaining interindividual differences in memory performance using 2 linear model comparisons. The first model comparison revealed that adding reinstatement as a predictor improved the model fit on memory performance (*R^2^* = 0.41) as compared with recognition specificity (*R^2^* = 0.24; *F*(64) = 9.64, *p* < 0.001). However, the second model comparison revealed that adding recognition specificity did not improve the model fit on *Pr* (*R^2^* = 0.41) as compared with reinstatement (*R^2^* = 0.39; *F*(64) = 1.21, *p* = 0.31). These findings suggest that between-participant variability in memory performance is best explained by category-level reinstatement as compared with recognition category specificity.

### Encoding-related category specificity explains interindividual variability in memory performance better than both recognition category specificity and category-level reinstatement

Here, we asked whether encoding category specificity was more functionally relevant for memory performance compared with recognition category specificity and category-level reinstatement using a series of model comparisons. First, we found that adding encoding category specificity as a predictor of memory performance improved the model fit (*R^2^* = 0.48) as compared with recognition category specificity and category-level reinstatement (*R^2^* = 0.41; *F*(62) = 5.56, *p*= 0.006). Next, we found that adding either recognition category specificity or category-level reinstatement did not improve the model fit on memory performance (respectively, *R^2^* = 0.46 and *R^2^* = 0.48) compared with encoding category specificity alone (*R^2^* = 0.46; *F*(64) = 0.73, *p* = 0.48 and *F*(64) = 2.22, *p* = 0.12, respectively). In sum, encoding category specificity tracked individual differences in memory performance better than both recognition category specificity and category-level reinstatement.

### Intraindividual variability in memory performance covaries with trial-wise category specificity

In the following text, we examined whether trial-wise category specificity predicted memory outcomes for each cluster identified during the searchlight similarity analyses across the whole sample. Using generalized linear mixed models, we predicted memory outcome (hit or miss) from age, category specificity, response bias, and the interaction between age and category specificity. A main effect of response bias was identified in every model (*p* < 0.001; see Table 4 for complete results). During encoding, a main effect of category specificity for faces was identified in the middle temporal and fusiform gyri cluster, as well as in the left hippocampus, indicating that higher category specificity was predictive of hits. These 2 clusters also revealed an interaction between age and category specificity, such that older adults revealed an attenuated relationship between category specificity and memory outcome (middle temporal gyrus: *M*_hits_ = 0.014, *M*_misses_ = 0.011; left hippocampus: *M*_hits_ = 0.004, *M*_misses_ = 0.004) compared with younger adults (middle temporal gyrus: *M*_hits_ = 0.020, *M*_misses_ = 0.011; left hippocampus: *M*_hits_ = 0.004, *M*_misses_ = 0.000). A main effect of category-level reinstatement for faces was also found in the fusiform and inferior occipitotemporal gyri cluster, with higher category specificity more likely resulting in a hit outcome. Finally, a main effect was found for both retrieval-related specificity and reinstatement for houses in the middle occipital and parahippocampal gyri clusters, again revealing that higher specificity was associated with a higher probability of hit. The models also revealed an age-related interaction in these clusters, such that the relationship between specificity and memory was attenuated in older adults (retrieval: *M*_hits_ = 0.024, *M*_misses_ = 0.024; reinstatement: *M*_hits_ = 0.013, *M*_misses_ = 0.015) compared with younger adults (retrieval: *M*_hits_ = 0.037, *M*_misses_ = 0.028; reinstatement: *M*_hits_ = 0.025, *M*_misses_ = 0.020). No other main effects or interactions reached significance.

**Table 4:** Results of generalized linear mixed effects models predicting trial-wise recognition memory accuracy from category specificity.

		*z*-values			
Searchlight	Regions	Age	RespBias	CatSpec	Age^*^CatSpec
**Face** **Encoding**	Middle temporal gyrus, fusiform gyrus, precuneus	−0.40	9.30^*^	4.09^*^	−2.54^*^
***(Category)***	Medial orbitofrontal gyrus, rectus	−0.91	8.61^*^	1.27	−1.30
	Hippocampus, putamen, amygdala, pallidum, fusiform gyrus	−0.98	8.76^*^	2.12^*^	−2.16^*^
	Amygdala, hippocampus, putamen, pallidum	−0.96	8.63^*^	1.68	−0.48
**Face** **Retrieval** ***(Category)***	Middle temporal gyrus, superior temporal gyrus, fusiform gyrus, inferior occipital gyrus, inferior temporal gyrus	−1.12	8.38^*^	−0.77	0.84
	Fusiform gyrus, inferior temporal gyrus, inferior occipital gyrus	−0.99	8.41^*^	−0.82	1.13
	Rectus, medial orbitofrontal gyrus	−0.94	8.39^*^	1.35	−0.86
	Middle temporal gyrus, superior temporal gyrus	−0.98	8.36^*^	−0.11	0.47
**Face Reinstatement** ***(Category)***	Middle temporal gyrus, fusiform gyrus, inferior temporal gyrus, inferior occipital gyrus, superior temporal gyrus	−0.98	9.42^*^	1.09	−0.35
	Fusiform gyrus, inferior occipital gyrus, inferior temporal gyrus	−0.84	8.91^*^	3.20^*^	−1.26
**House** **Encoding**	Middle occipital gyrus, precuneus	−1.00	9.56^*^	1.77	−0.42
***(Category)***	Caudate	−1.08	9.31^*^	0.07	−0.86
**House** **Retrieval** ***(Category)***	Middle occipital gyrus, precuneus, parahippocampal gyrus	−0.09	9.13^*^	2.69^*^	−3.02^*^
**House Reinstatement** ***(Category)***	Middle occipital gyrus, lingual gyrus, precuneus, parahippocampal gyrus	0.69	9.16^*^	3.41^*^	−2.66^*^
	Middle cingulate cortex	−1.20	9.46^*^	0.90	−1.50

^^*^^ *p* < 0.05.

### Intraindividual variability in memory performance covaries with item-level reinstatement in calcarine cortex

In the final step, we were interested in whether the ability to reinstate item- or category-level encoding information or hippocampal activity during retrieval were associated with within-person memory outcomes. Our item-level reinstatement searchlight similarity analysis for houses revealed 2 significant clusters: one primarily located in the calcarine cortex and the other located in and around the fusiform cortex. For each cluster, we performed a generalized linear mixed effects model in order to test whether binary memory response outcome (hit or miss) could be predicted by response bias, trial-wise item reinstatement, category reinstatement (within the item reinstatement clusters), or retrieval-related hippocampal activity. We found that trial-wise item reinstatement predicted memory outcome in the calcarine cluster (log odds = 2.40, 95% CI [1.29–4.43]), but not in the fusiform cluster (log odds = 1.71, 95% CI [0.87–3.36]). We additionally found that response bias predicted memory outcome in both models (calcarine cluster: log odds = 73.49, 95% CI [33.58–160.85]; fusiform cluster: log odds = 75.35, 95% CI [35.55–159.74]). No other fixed effects or interactions reached significance (see Table 5).

**Table 5:** Results of generalized linear mixed effects models predicting trial-wise recognition memory accuracy from item-level reinstatement and hippocampal activity.

Cluster	Fixed effects predictors	Log odds	*z*	*p*	95% CI lower	95% CI upper
**Calcarine (*k* = 196)**	Age	0.91	−0.90	0.37	0.73	1.12
	**Response Bias**	**73.49**	**10.75**	**<0.001**	**33.58**	**160.85**
	Hippocampal activity	1.00	−0.06	0.95	0.90	1.10
	**Item reinstatement**	**2.40**	**2.77**	**0.006**	**1.29**	**4.43**
	Category reinstatement	3.83	0.92	0.36	0.22	67.61
	Age ^*^ hippocampal activity	1.00	0.00	1.00	0.87	1.16
	Age ^*^ item reinstatement	0.42	−1.74	0.08	0.16	1.12
	Age ^*^ category reinstatement	0.18	−0.79	0.43	0.00	12.28
**Fusiform (*k* = 83)**	Age	0.87	−1.35	0.18	0.71	1.07
	**Response Bias**	**75.35**	**11.28**	**<0.001**	**35.55**	**159.74**
	Hippocampal activity	1.00	−0.03	0.98	0.90	1.10
	Item reinstatement	1.71	1.57	0.12	0.87	3.36
	Category reinstatement	1.26	0.21	0.84	0.14	11.25
	Age ^*^ hippocampal activity	1.00	−0.07	0.95	0.86	1.15
	Age ^*^ item reinstatement	0.74	−0.59	0.56	0.26	2.05
	Age ^*^ category reinstatement	0.80	−0.12	0.90	0.02	28.42

CI = confidence interval; significant effects are shown in bold.

## Discussion

This study implemented exploratory multivariate pattern similarity searchlight analyses in order to investigate the influence of age-related neural dedifferentiation on category-sensitive neural representations during memory encoding, retrieval, and encoding–retrieval reinstatement and the relationships to memory performance. We used data from a memory paradigm completed in the fMRI scanner in which younger and older adults incidentally learned face and house stimuli and subsequently completed a surprise old/new recognition test. In line with the literature on age differences in memory performance using recognition paradigms (e.g. Craik and McDowd 1987), corrected recognition scores did not differ between age groups. However, age differences were evident in both hit rate and false alarm rate, with older adults responding “old” more often both to old and new stimuli, suggesting that older adults had reduced memory specificity compared with younger adults (Jacoby and Rhodes 2006; Fandakova et al. 2018). On the neural level, distinctiveness, as reflected in less similar neural representations of items between different stimulus categories compared with items within the same stimulus category, was observed during all memory phases (encoding, reinstatement, and retrieval) for both face and house stimuli. Importantly, younger adults demonstrated more distinctive representations than older adults, in line with the age-related neural dedifferentiation hypothesis (Li et al. 2001; for review, see Koen and Rugg 2019). Interindividual differences in neural distinctiveness during retrieval and reinstatement were associated with encoding-related variability in distinctiveness. Finally, the distinctiveness of categorical representations was linked to better memory performance both on a within-person level as well as across individuals. In sum, our results contribute to understanding how age-related neural dedifferentiation presents across multiple memory phases as well as how neural distinctiveness relates to memory performance.

First and foremost, this study adds to the scant literature on age-related neural dedifferentiation during memory retrieval. While many studies have highlighted the importance of representational distinctiveness during encoding (Zheng et al. 2018; Koen et al, 2019; Srokova et al. 2020; Hill et al. 2021), and encoding–retrieval reinstatement (St-Laurent et al. 2014; Hill et al. 2021), the question of how age impacts neural distinctiveness during memory retrieval has been less discussed. Studies providing evidence of representational transformation between encoding and retrieval (Favila et al. 2020) led us to hypothesize that neural distinctiveness may decline differentially between these 2 memory phases. Additionally, in a recent review (Sander et al. 2021), we argued that lower representational distinctiveness during encoding might increase the demands on retrieval monitoring processes, which have also been shown to be affected by aging (Fandakova et al. 2013), suggesting that age differences might accumulate across memory phases. Here, we found age differences in neural distinctiveness across all memory phases, which were primarily located in ventral visual cortices (see also Koen and Rugg 2019). In line with prior studies (Johnson et al. 2015; Hill et al. 2021), age differences in both retrieval- and reinstatement-related distinctiveness could be mostly explained by age differences already evident during encoding. These findings suggest that the observed age differences in distinctiveness during retrieval likely reflect a recapitulation of the (poorly) encoded representations or poor perceptual (re-)processing rather than an accumulation of processing deficiencies across memory phases.

Theories of cognitive aging (S-C Li and Lindenberger 1999; S-C Li et al. 2001; for reviews, see S-C Li and Rieckmann 2014; Koen and Rugg 2019) hypothesize that neural dedifferentiation impairs memory performance. Several studies have recently demonstrated a positive relationship between neural distinctiveness during encoding and memory performance (Zheng et al. 2018; Koen et al, 2019; Srokova et al. 2020) and between reinstatement and memory performance (St-Laurent et al. 2014; Abdulrahman et al. 2017; Bowman et al. 2019; Hill et al. 2021). We sought to perform a comprehensive analysis by relating interindividual differences in memory performance to neural distinctiveness across multiple memory phases. Crucially, higher neural distinctiveness during retrieval was associated with better memory performance across individuals. However, compared with retrieval, reinstatement-related distinctiveness was far better at predicting memory performance. This aligns with several previous aging studies also reporting the beneficial impact of precise reinstatement on memory performance (St-Laurent et al. 2014; Abdulrahman et al. 2017; Bowman et al. 2019; Hill et al. 2021; but see Wang et al. 2016). Finally, encoding-related distinctiveness explained variability in memory performance to an even greater extent than both retrieval- and reinstatement-related distinctiveness, underlining the importance of representing information distinctively during the initial experience. Together, these results provide clear evidence that interindividual differences in memory performance are related to differences in neural distinctiveness.

In addition to interindividual relationships, trial-by-trial variations in representational distinctiveness have been suggested to further influence recognition success. This hypothesis was supported by our trial-wise analyses. Specifically, we found that higher neural distinctiveness of a given face or house trial resulted in a higher probability of a hit outcome. For example, category-level encoding- and reinstatement-related distinctiveness were predictive of memory outcome for faces, and retrieval- and reinstatement-related distinctiveness for houses (see Kuhl et al. 2012; Gordon et al. 2014; Trelle et al. 2020; Hill et al. 2021 for similar findings). Interestingly, we observed age differences in several of these relationships, with younger adults demonstrating a stronger relationship between distinctiveness and memory than older adults. This outcome resembles findings reported by Hill et al. (2021), who also showed an analogous age-moderated relationship between trial-wise category-level reinstatement and source memory performance for scene stimuli. Thus, trial-to-trial distinctiveness may not track memory outcomes as readily in older adults compared with younger adults. One caveat to this conclusion is that older adults tended to respond “old” more often than younger adults regardless of whether the stimulus was actually old or new, indicating that their hits are a combination of genuinely recollected trials and correctly guessed trials. Although we tried to control for this by including the response bias in our models, this confound may nevertheless impact the accuracy of our models for older adults.

While we found age differences in neural distinctiveness only on the category level, age-related neural dedifferentiation has also been reported in terms of a decrease in the distinctiveness of item-specific reinstatement (Folville et al. 2020; Hill et al. 2021). Although we observed occipital regions demonstrating an effect of strong item-level distinctiveness during reinstatement for houses, we found neither age differences in this effect nor a relationship to interindividual differences in memory performance. The absence of age differences in item-level reinstatement is surprising and appears to contradict previous evidence for an age deficit at this representational level. However, while Hill et al. (2021) demonstrated an age-related decrease in item-level pattern similarity, their measure did not control for potential age deficits at the category level. Therefore, the observed age differences at the item level may not have reflected more than a general categorical deficit. Nevertheless, our finding proves difficult to interpret in the context of the current literature—more studies will be needed to understand how age differences in neural distinctiveness vary across different representational levels.

The use of a recognition-based retrieval paradigm (as opposed to source memory or cued recall paradigm) may have some impact on the findings of this study. First of all, active perceptual input during retrieval makes it difficult to definitively disentangle whether neural dedifferentiation during retrieval is related to poorly reinstated encoding patterns or poor perceptual representations of the to-be-recognized item or some combination of both. (However, our observation that trial-wise variations in neural distinctiveness related to memory outcomes indicates that these retrieval-related patterns have at least some mnemonic relevance.) Second, the absence of age differences in item-level reinstatement could indicate that the active perceptual input during retrieval supported representational distinctiveness in older adults in lieu of poorly reinstated encoding representations (see Folville et al. 2020; Hill et al. 2021 for age differences in item-specific reinstatement). Finally, in contrast to prior studies (Trelle et al. 2020; Hill et al. 2021), we did not find that retrieval-related hippocampal activity predicted memory success, possibly suggesting this retrieval paradigm did not recruit the hippocampus to the same extent as other retrieval paradigms might (but, see Ritchey et al. 2013 for evidence of hippocampal recruitment during a recognition task). In sum, further work is needed to understand how retrieval tasks might modulate manifestations of age-related neural dedifferentiation and hippocampal activation during retrieval.

Together, our findings reveal evidence for age-related neural dedifferentiation during memory encoding, retrieval, and encoding–retrieval reinstatement and provide support for a link between neural distinctiveness and memory performance both on an individual trial level and across individuals. Importantly, the observed age deficits in neural distinctiveness during retrieval and reinstatement were closely tied to the age deficit during encoding. Higher neural distinctiveness across all memory phases was associated with better memory performance with encoding-related distinctiveness explaining the most interindividual variability in memory compared with retrieval and reinstatement. Together, our results highlight the importance of processing information distinctively upon initial perception and throughout all memory stages.

## Supplementary Material

Supplements_bhad219

## CRediT author Statement

Claire Pauley (Data curation, Formal analysis, Methodology, Software, Visualization, Writing—original draft, Writing—review & editing), Malte Kobelt (Methodology, Software, Writing—review & editing), Markus Werkle-Bergner (Conceptualization, Funding acquisition, Project administration, Writing—review & editing), Myriam C. Sander (Conceptualization, Funding acquisition, Methodology, Project administration, Supervision, Writing—review & editing).

## Resource availability

Scripts are publicly available on osf: https://osf.io/rm65w/.
