# Supplementary material for: Age differences in neural distinctiveness during memory encoding, retrieval, and reinstatement
Source: Cereb Cortex. 2023 Jun 26;33(16):9489–503. doi: 10.1093/cercor/bhad219 (PMC10431749; doi:10.1093/cercor/bhad219)
Supplement: Supplements_bhad219 [file supplements_bhad219.docx]

**Running searchlight analyses independently for younger and older adults**

Our reported searchlight analyses were initially computed across age groups and then looked for age differences within these maps. There are a couple of reasons we decided to use this method (as opposed to looking into younger adults alone). One reason is that this method is an age-fair comparison that takes younger and older adults equally into consideration and does not bias our subsequent analysis for age differences in one direction or the other. Furthermore, this method increases the power behind these contrasts by including more participants, which helps increase the signal-to-noise ratio and actually leads to slightly larger regions coming out of the analyses in most cases (see Figure S1).


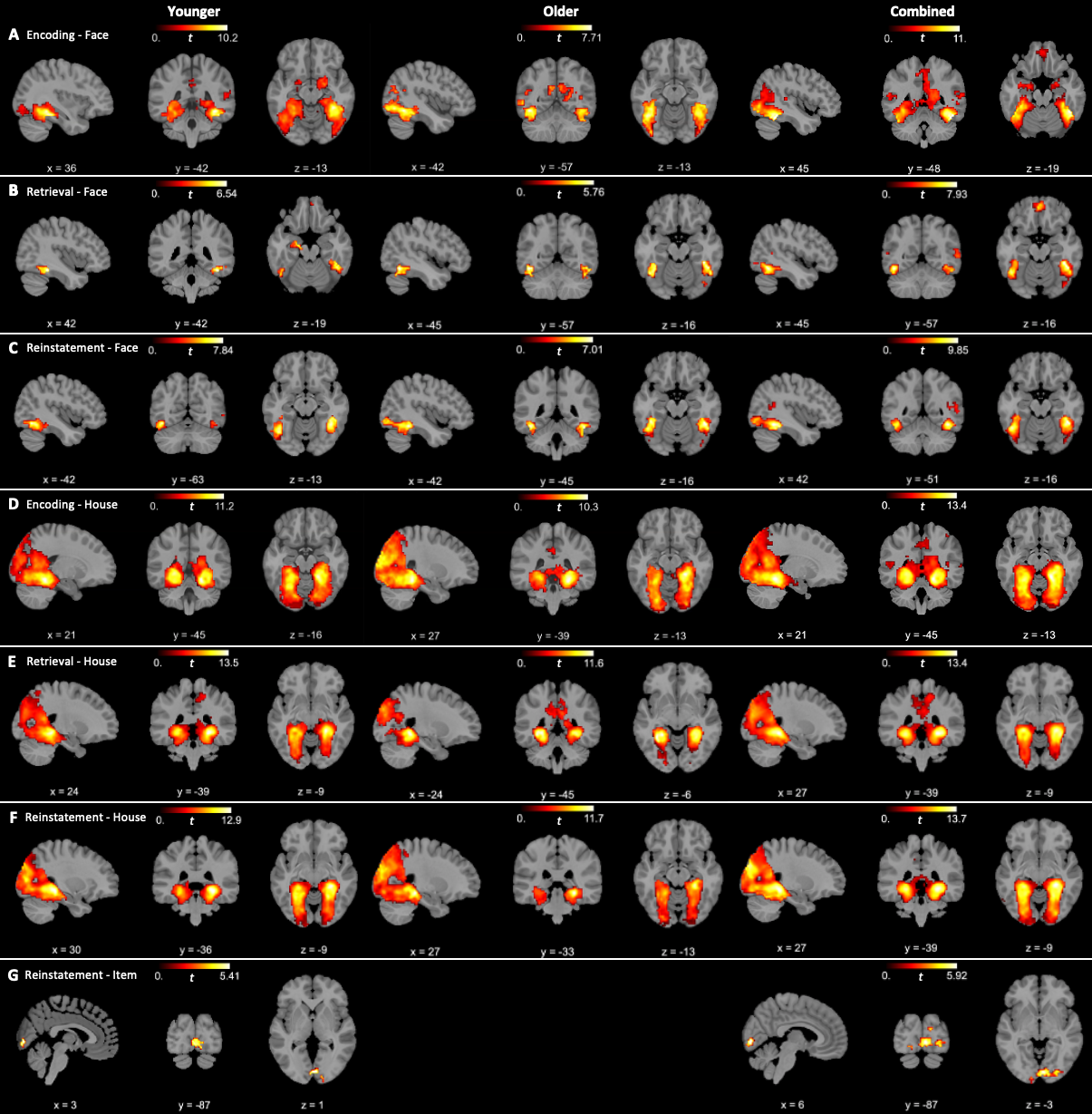


Figure S1. Regions demonstrating category specificity during encoding, recognition and reinstatement for both faces (A, B, C) and houses (D, E, F) in younger adults (left column), older adults (middle column), and combined age group (right column). Regions demonstrating item-level reinstatement specificity for houses (G).

**Separating reinstatement effects by first and second encoding run**

Our paradigm consisted of two encoding runs, in which the same stimuli were presented in each run, followed by an old/new recognition task. In the manuscript, we report encoding-retrieval reinstatement based on similarity values averaged across the two encoding runs. At the request of a reviewer, we separated the analyses by encoding run as can be seen in Figure S2. The findings show large agreement for category-level reinstatement specificity for both faces and houses between reinstatement of the encoding patterns from the first and second encoding runs. No item-level specificity was found for faces or houses using the first and second encoding runs alone for the reinstatement searchlight analysis (*p*s > 0.06). This is likely due to cutting the number of trials for each searchlight contrast in half. Averaging across the reinstatement of first and second encoding likely increases the signal-to-noise ratio of the fine-grained item-specific patterns.


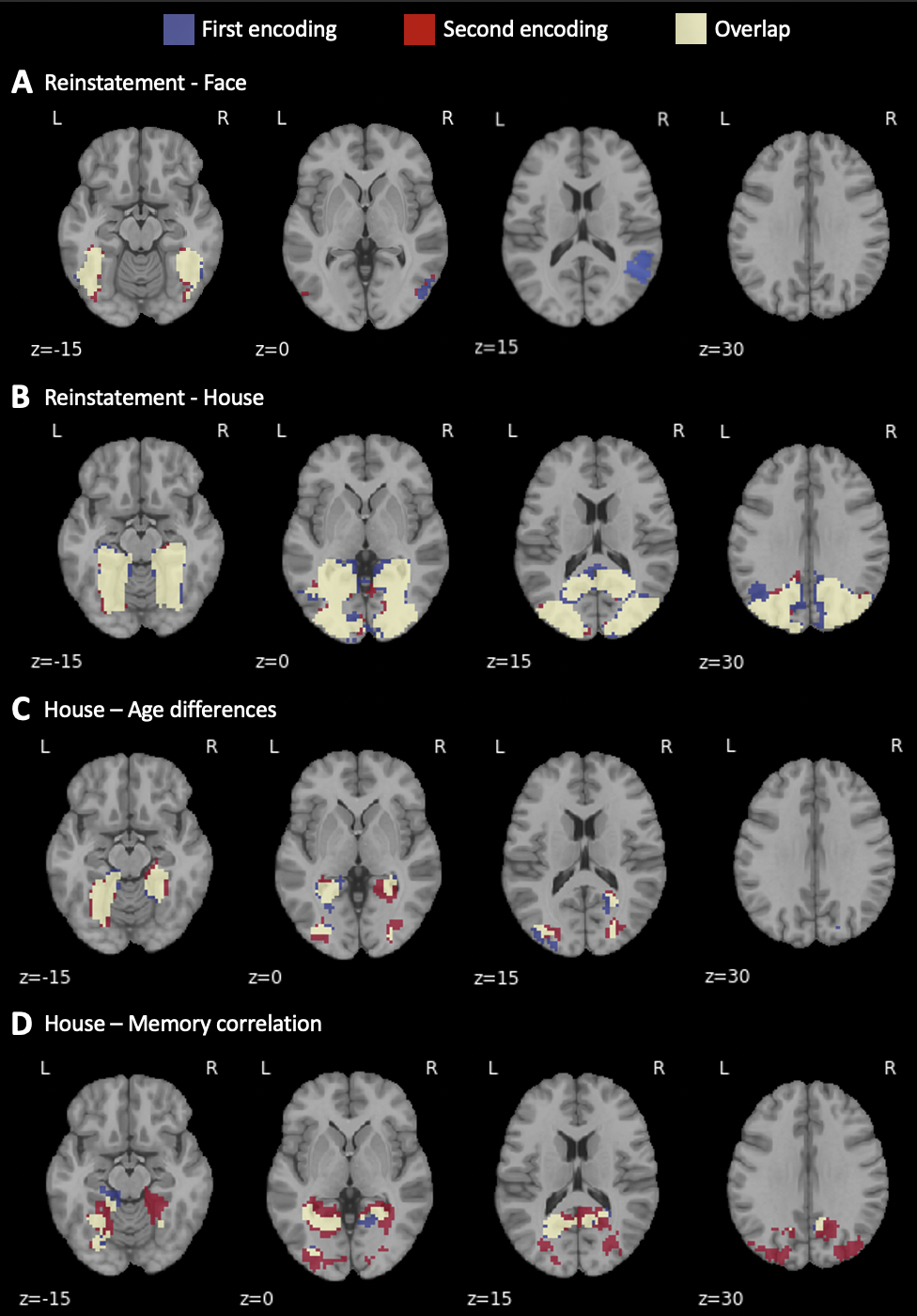


Figure S2. Voxels demonstrating category-level reinstatement between the first encoding run and recognition and between the second encoding run and recognition for faces (A) and houses (B). Blue = voxels only identified during reinstatement of first encoding patterns; red = voxels only identified during reinstatement of second encoding patterns; yellow = voxels in agreement between reinstatement of first and second encoding patterns.
